# Complete chloroplast genome sequences of *Lagotis brevituba* (Plantaginaceae): a famous Tibetan medicine plant

**DOI:** 10.1080/23802359.2021.1927216

**Published:** 2021-05-17

**Authors:** Cheng-Xian Fan, Ying-Min Zhang, Shi-Biao Pu, Guo-Dong Li

**Affiliations:** a Yunnan Key Laboratory for Dai and Yi Medicines, Yunnan University of Chinese Medicine, Kunming, Yunnan, China; b Faculty of Traditional Chinese Pharmacy, Yunnan University of Chinese Medicine, Kunming, Yunnan, China

**Keywords:** *Lagotis brevituba*, chloroplast genome, phylogenomic analysis, Tibet medicinal plant

## Abstract

*Lagotis brevituba* is a famous Tibetan medicine plant and its complete chloroplast genome is determined in this study. The complete chloroplast genome is 152,967 bp in length, with a large single-copy (LSC) region of 83,740 bp, a small single copy (SSC) region of 17,845 bp, and a pair of inverted repeats (IRs) of 25,691 bp. The whole genome contained 131 genes, including 86 protein-coding genes, 37 tRNA genes and 8 rRNA genes. The phylogenetic tree showed that *L. brevituba* clustered with *L. yunnanensis* in family Plantaginaceae.

*Lagotis brevituba* Maxim. is a perennial species that grows in mountainous regions at altitudes in the range of 3800–4800 m (Angiosperm Phylogeny Group 2016). This plant is endemic to the Qinghai-Tibetan plateau and has been used for centuries in traditional Tibetan medicine for the treatment of acute and chronic hepatitis, hypertension, nephritis and breast cancer (Editorial Committee of Tibetan Materia Medica 1991; Zhu et al. 2017). Here, we sequenced, assembled, and annotated the whole chloroplast genome of *L. brevituba* and constructed the phylogenetic tree to determine its phylogenetic relationship between and within related species.

Fresh leaves of *L. brevituba* were sampled from Yushu, Qinghai, China (33°8′N, 96°43′E). The voucher specimen was deposited at the Herbarium of Yunnan University of Chinese Medicine (Guo-Dong Li, gammar116@163.com) under the voucher number 5334211276. The total genomic DNA was extracted using plant DNA (Bioteke Corporation, Beijing, China). A library was constructed and sequencing was performed on an Illumina HiSeq 2500 platform (Illumina Inc, SanDiego, CA). All raw reads were performed with NGS QC Toolkit (Patel and Jain 2012). The filtered reads were assembled using NOVOPlasty (Dierckxsens et al. 2017) with complete genome of its close relative *L. yunnanensis* (MN752238) as the reference. The assembled chloroplast genome was annotated using Geseq (Tillich et al. 2017) and the annotated genome was corrected using Generous R11 11.1.5 (Biomatters Ltd., Auckland, New Zealand).

The complete chloroplast genome of *L. brevituba* (GenBank accession no. MW182582) was 152,967 bp in length, with a large single-copy (LSC) region of 83,740 bp, a small single-copy (SSC) region of 17,845 bp, and a pair of inverted repeats (IRs) regions of 25,691 bp each. The whole genome contained 131 genes, including 86 protein-coding genes, 37 tRNA genes, and 8 rRNA genes. The overall GC content of the whole plastome, LSC, SSC, and IR regions is 38.3%, 36.5%, 32.5%, 43.2%, respectively. A total of 57 SSRs were detected using the online software MISA (Beier et al. 2017). The number of mono-, di-, tri-, tetra-, penta-, and hexa- nucleotides SSRs are 33, 7, 8, 4, 2, and 3, respectively.

To explore the phylogenetic position of *L. brevituba* among the limited number of species available across *Lagotis*, complete cp genomes of 16 species within Scrophulariaceae and Plantaginaceae were selected to conduct analyses, using *Pedicularis ishidoyana* and *Orobanche californica* from Orobanchaceae as outgroups. Multiple sequence alignments of cp genome sequences were performed using MAFFT v.7 (Katoh and Standley 2013). The RAxML inference was performed by using the GTR model with support for branches evaluated by 1000 bootstrap replicates (Stamatakis 2014). The phylogenetic tree showed that *L. brevituba* clustered with *L. yunnanensis* in family Plantaginaceae (Figure 1). This genetic information will contribute to phylogenomic study of the genus Lagotis in the future.

**Figure 1.**
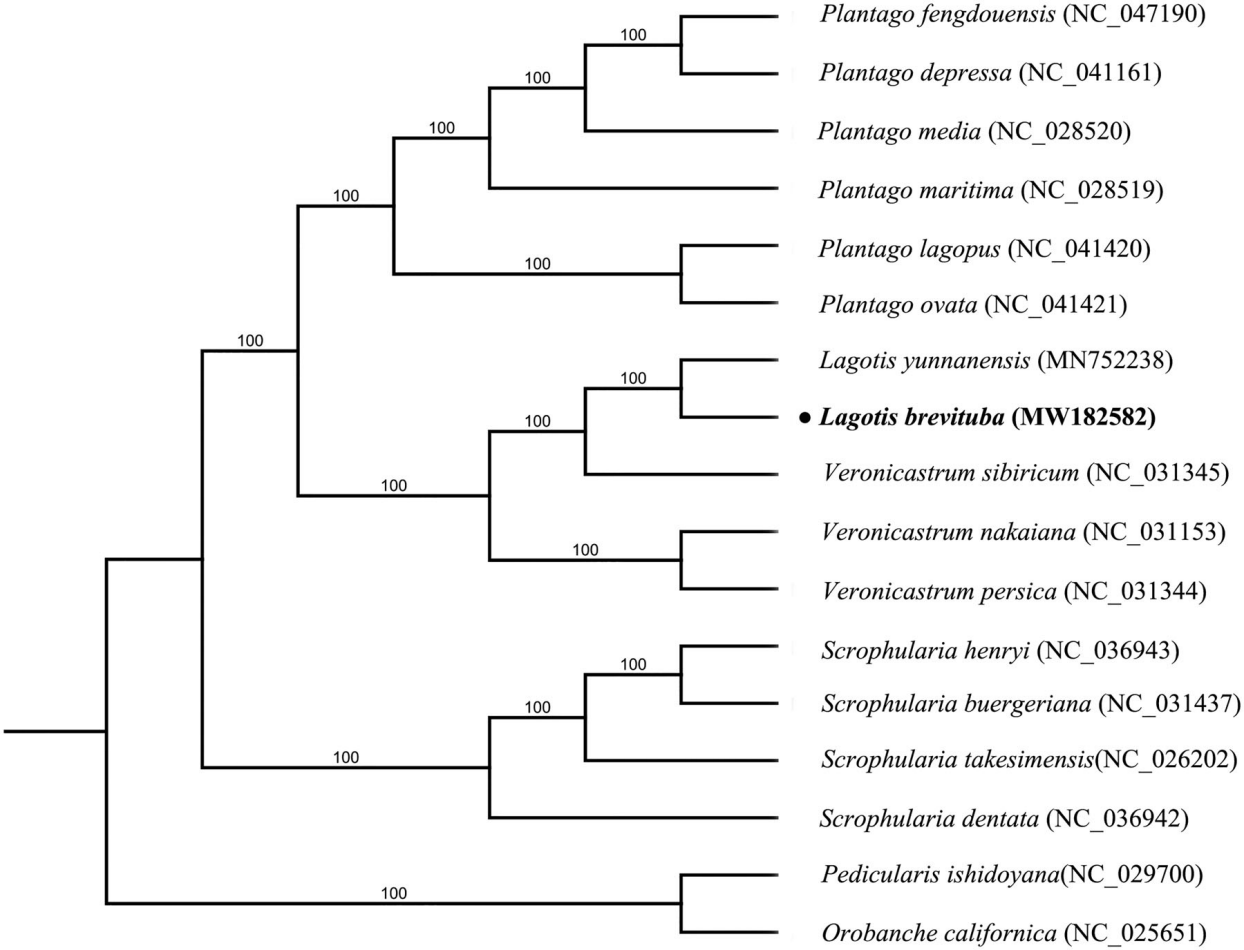
Maximum-likelihood phylogenetic tree inferred from 17 chloroplast genomes. Bootstrap support values >50% are indicated next to the branches.

## Data Availability

The *Lagotis brevituba* data that support the findings of this study are openly available in GenBank of NCBI at https://www.ncbi.nlm.nih.gov under the Accession no. MW-182582. The associated BioProject, SRA, and Bio-Sample numbers are PRJNA720711, SR14226810, and SAMN18677722, respectively.
